# Use of linked electronic health records to assess mortality and length of stay associated with pandemic influenza A(H1N1)pdm09 at a UK teaching hospital

**DOI:** 10.1017/S0950268814002076

**Published:** 2014-08-14

**Authors:** C. Smith, M. D. Curran, I. Roddick, M. Reacher

**Affiliations:** 1 Field Epidemiology Services – Cambridge, Public Health England, Cambridge, UK; 2 Department of Infection and Population Health, Centre for Infectious Disease Epidemiology, University College London, London, UK; 3 Clinical Microbiology and Public Health Laboratory, Public Health England, Addenbrooke's Hospital, Cambridge, UK

**Keywords:** Infectious disease epidemiology, influenza A

## Abstract

Effective use of data linkage is becoming an increasingly important focus in the new healthcare system in England. We linked data from the results of a multiplex PCR assay for respiratory viruses for a population of 230 inpatients at a UK teaching hospital with their patient administrative system records in order to compare the mortality and length of stay of patients who tested positive for influenza A(H1N1)pdm09 with those positive for another influenza A virus. The results indicated a reduced risk of death among influenza A(H1N1)pdm09 patients compared to other influenza A strains, with an adjusted risk ratio of 0·25 (95% confidence interval 0·08–0·75, *P* = 0·01), while no significant differences were found between the lengths of stay in the hospital for these two groups. Further development of such methods to link hospital data in a routine fashion could provide a rapid means of gaining epidemiological insights into emerging infectious diseases.

Linkage of electronic health records to connect information across disparate sources can represent a valuable tool for medical and population health research [1]. The importance of exploiting such tools has been highlighted numerous times during the development of the new healthcare system in England: Public Health England included improved data sharing as one of its key priorities for strengthening public health surveillance [2], and NHS England and the Health and Social Care Information Centre are aiming to link data for patients across health and social care systems through the development of the *care.data* service [3]. However, the process of linking datasets that have not been designed with this objective in mind can be complex, as there is not always a consistent unique identifier that can be used to match a record from one database to a corresponding record for the same patient (or encounter, depending on the purpose), in another database. Data structures often differ, requiring multiple entries to be matched to a single row in another database, and completeness of key linking fields is not always prioritized in data entry.

When the pandemic influenza A(H1N1)pdm09 virus emerged in 2009, uncertainty regarding its nature and severity prompted efforts to rapidly determine the burden of mortality and morbidity associated with the strain [4]. In this study, we aimed to demonstrate how linkage of routinely collected hospital data could be used to address this question and similar and population-level concerns.

This was a retrospective study of a group of inpatients at a UK teaching hospital who tested positive for an influenza A virus by multiplex PCR between November 2007 and December 2012. Laboratory multiplex PCR results were linked to their results in a second genotyping database to determine viral subtypes. Corresponding records were also identified in the Patient Administration System (PAS), allowing identification of inpatients, the reason for their admission, discharge destination, and admission and discharge dates. Microsoft SQL Server 2005 (Microsoft Corp., USA) was used to perform the linkage, based on patients' hospital numbers, names and hospital spell identification numbers (an identifier for the continuous stay of a patient in a hospital). Deaths were identified using the discharge destination field, and length of stay following a positive influenza A test was calculated using the testing and discharge dates.

The multiplex PCR assay for respiratory viruses used at this hospital includes a pentaplex influenza A/B/A(H1N1)pdm09/H3 detection and typing real-time assay with an internal control, which has been detailed previously [5, 6], with additional multiplex real-time (Taqman) PCR assays to detect other common respiratory viruses including respiratory syncytial viruses (RSV) A and B, parainfluenza types 1–4, adenovirus, enterovirus, rhinovirus, human metapneumovirus, group 1 coronaviruses (HCoV-229E and HCoV-NL63), group 2 coronaviruses, and SARS-associated coronavirus [7].

Distributions of mortality and length of stay following a positive influenza A test were compared for (H1N1)pdm09 and other influenza A cases, and relationships assessed using univariable and multivariable log-binomial regression analyses as appropriate.

The study population comprised 243 patients who tested positive for influenza A, from a total of 4895 inpatients who had a multiplex respiratory PCR test during their admission between November 2007 and December 2012. Successful linkages between the multiplex PCR, genotype, and PAS databases were obtained for all records with the exception of 13 patients who tested positive for influenza A but for whom no genotyping results were available and were therefore excluded from the analysis. Linkages were achieved based on the combination of hospital numbers and hospital spell identifiers and checked manually using names. A total of 160 (70%) of the 230 included patients had influenza A(H1N1)pdm09, with the remaining 70 testing positive for another influenza A strain, including 62 H3 and eight non-pandemic H1 isolates which typed as the seasonal H1N1 strain circulating prior to and during the early phase of the pandemic in 2009.

Five influenza seasons were included in the data. The pandemic H1N1(pdm09) strain first arose in the 2008/2009 season, and predominated for the next two seasons, until 2011/2012 in which no H1N1 cases were detected.

About half (118, 51%) of the patients were male and the median age was 34·5 years [inter-quartile range (IQR) 10–67·5 years]. The majority of the patients (182, 79%) were admitted for a respiratory reason, and 32 (14%) tested positive for at least one other respiratory virus. Of these, five had one co-infection, 26 had two co-infections and one had three co-infections; the most common co-infecting viruses were rhinovirus and RSV (11 and 10 patients, respectively).

A total of 13 patients in the population died while in hospital. These fatalities included four (2·5%) of the 160 patients with H1N1 influenza A, and nine (13%) of the 70 patients with non-H1N1 influenza A. The results of the univariable and multivariable log-binomial regression analyses for the outcome of death are shown in Table 1. The crude risk ratio (RR) for death following an infection with influenza A(H1N1)pdm09 compared to other influenza A viruses was 0·19 [95% confidence interval (CI) 0·06–0·61, *P* < 0·01]. A larger proportion of patients aged ⩾65 years died, with a RR of 16·5 (95% CI 2·17–126·2, *P* < 0·01) compared to the baseline group (those aged 0–16 years). There were non-significant associations between male sex and reduced risk of death (RR 0·42, 95% CI 0·13–1·33, *P* = 0·13), and between presence of a co-infection and increased risk of death (RR 1·13, 95% CI 0·26–4·84, *P* = 0·88). None of the 48 patients who were admitted for a non-respiratory reason died.

**Table 1.** Univariable and multivariable analyses for effect of influenza A strain type on risk of death among inpatients testing positive for influenza A at a UK teaching hospital, November 2007 to December 2012

		Total	Died		Univariable analysis			Multivariable analysis		
Variable	Level	(*N* = 230)	(*N* = 13)	%	RR	95% CI	*P*	aRR	95% CI	*P*
Influenza A virus	Non-H1N1*	70	9	12·85	Base	—	—	—	—	—
	(H1N1)pdm09	160	4	2·50	0·19	0·06–0·61	<0·01	0·25	0·08–0·75	0·01
Age, years	0–16	62	1	1·61	Base	—	—	—	—	—
	17–64	138	4	2·90	1·80	0·21–15·75	0·60	2·34	0·27–20·60	0·44
	⩾65	30	8	26·67	16·5	2·17–126·22	<0·01	14·09	1·83–108·43	0·01
Sex	Female	112	9	8·04	Base	—	—	—	—	—
	Male	118	4	3·39	0·42	0·13–1·33	0·13	0·58	0·21–1·60	0·29
Co-infection†	No	198	11	5·56	Base	—	—	—	—	—
	Yes	32	2	6·25	1·13	0·26–4·84	0·88	—	—	—
Reason for admission	Respiratory	182	13	7·14	—	—	—	—	—	—
	Non-Respiratory	48	0	0	—	—	—	—	—	—

RR, Risk ratio; CI, confidence interval; aRR, adjusted risk ratio (multivariable log-binomial regression of effect of influenza A strain type on risk of death, adjusted for age and sex)

* Non-H1N1, any influenza A virus except (H1N1)2009

† Co-infection, positive test for any other respiratory virus by multiplex PCR

Multivariable analysis was performed adjusting for age, as a significant covariate, and sex, as an *a priori* confounder, but not co-infection or reason for admission as they were not significantly associated with death at univariable analysis. The adjusted RR for influenza A(H1N1)pdm09 increased to 0·25 (95% CI 0·08–0·75, *P* = 0·01), which suggests that the crude RR was partially confounded by these factors.

The median lengths of stay in the hospital following a positive test for influenza A among patients with influenza A(H1N1)pdm09 and other influenza A viruses were 3 days (IQR 1–9 days) and 4 days (IQR 2–12 days), respectively. Inspection of the distributions did not suggest any differences between these groups, and a Wilcoxon–Mann–Whitney test confirmed that the distributions were not significantly different (*P* = 0·32).

This study was the first analysis of linked multiplex respiratory virus PCR and patient administration system data from this hospital. The successful linkage of these datasets allowed the detailed virological information that had been collected over five influenza seasons to be enriched with demographic and clinical data in order to address this important epidemiological problem.

The results provided evidence that influenza A(H1N1)pdm09 was less severe in terms of mortality than other influenza A viruses, with a risk of death about one quarter that of seasonal influenza A. No significant differences were found in the lengths of stay in the hospital for these two groups. These results are consistent with other reports that the pandemic strain was less severe than initially anticipated [8], and did not result in as many deaths as other influenza pandemics in the 20th century [9, 10].

The case-fatality ratio (CFR) in this population for pandemic influenza A(H1N1)pdm09 was 2·5%. Since this study concerned hospitalized patients only, it is not surprising that this value exceeds some other reported values, such as the estimated CFR in the UK from the first year of the pandemic of 0·04% [11], which was calculated using estimated number of clinical cases from surveillance data as the denominator and number of deaths associated with influenza A(H1N1)pdm09 as the numerator. A recent review [12] identified a wide range of published CFRs from this pandemic, with higher estimates from hospitalized cases, and a tenfold increase in the CFR for those requiring intensive care compared to all symptomatic cases.

There were a number of limitations to this study which impact on its generalizability to other influenza A patient populations. First, although the reason for admission could be classified as respiratory or non-respiratory, there was not enough clinical information available to generate an accurate measure of underlying conditions in the patients, which therefore could not be controlled for in the analysis. Previous studies have indicated that factors such as pregnancy, immunosuppression and neurological disorders [13], are related to severe infection and would therefore be important to consider. Bacterial co-infections have also been associated with fatal cases of influenza A(H1N1)pdm09 infection [14], a finding that could be assessed through linkage with other routinely collected hospital laboratory data, although full reviews of clinical notes would be required to provide a complete understanding of the pre-existing medical conditions.

Second, the mortality outcome was calculated using only the patient discharge destination, and therefore captured only patients who died while in hospital. Linkage to death registers would enable better understanding of the possible effects of these virus strains on mortality, but this was beyond the scope of this study.

Third, the data collected were limited to inpatients at one teaching hospital across five influenza seasons. The study population is therefore not representative of the entire population of patients with influenza A across these seasons, as inpatients at this hospital are likely to have more severe disease than non-inpatients, and may have complicated pre-existing conditions.

Finally, there was a potential admission bias in this study: during the pandemic, public awareness of influenza was heightened, and, although public health advice recommended that symptomatic individuals stay at home [15], it is possible that there were relaxed admission standards during the first phase of the pandemic. More liberal admission criteria could have led to an increase in the number of non-severe cases admitted compared to other influenza seasons and therefore contributed to the observed association with reduced mortality. However, no formal policy to this effect was introduced at this hospital, and such a situation would also be likely to lead to a reduction in the average length of stay, which was not detected here.

In spite of these limitations, the association between influenza A(H1N1)pdm09 H1N1 and reduced mortality among influenza A patients was strong, and provides further evidence that this strain of influenza was not as severe as was first feared. Moreover, this analysis emphasizes the value of combining databases of electronic healthcare records to make the best use of routinely collected data. Further development of such data linkage methods in a routine fashion could provide a rapid means of gaining valuable epidemiological insights into emerging infectious diseases.
